# Folic Acid Prevents High-Fat Diet-Induced Postpartum Weight Retention in Rats, Which Is Associated with a Reduction in Endoplasmic Reticulum Stress-Mediated Hepatic Lipogenesis

**DOI:** 10.3390/nu16244377

**Published:** 2024-12-19

**Authors:** Huaqi Zhang, Li Zhang, Xuenuo Zhao, Yanzhen Ma, Dan Sun, Yixian Bai, Weiheng Liu, Xi Liang, Hui Liang

**Affiliations:** Department of Nutrition and Food Hygiene, School of Public Health, Qingdao University, 308 Ningxia Road, Qingdao 266071, China; huaqi_erin@163.com (H.Z.); zhangli200513@163.com (L.Z.); nuo0109996@163.com (X.Z.); mayanzhen97@163.com (Y.M.); sundan202409@163.com (D.S.); bai_yixian@163.com (Y.B.); 18648646729@163.com (W.L.); liangxi6029@163.com (X.L.)

**Keywords:** folic acid, endoplasmic reticulum stress, postpartum weight retention

## Abstract

Background: Proactively preventing postpartum weight retention (PPWR) is one of the effective intervention strategies to reduce the occurrence of obesity in women. Population studies have shown that serum folate levels are closely related to body weight. The regulation of folic acid on lipid metabolism has been fully confirmed in both in vivo and in vitro studies. For many years, folic acid supplementation has been widely used in periconceptional women due to its role in preventing fetal neural tube defects. However, whether folic acid supplementation prior to and throughout pregnancy exerts preventive effects on PPWR remains uncertain. This study aims to investigate the preventive effect of folic acid on PPWR in rats and further explore the underlying mechanisms. Methods: In this study, pregnant rats were administered one of the dietary schedules: control diet (CON), high-fat diet (HF), control diet combined with folic acid (FA) and high-fat diet combined with folic acid (HF + FA). Results: We discovered that folic acid supplementation inhibited high-fat diet-induced elevations in body weight, visceral fat weight, liver weight, hepatic lipid levels and serum lipid levels at 1 week post-weaning (PW). Western blot analysis showed that folic acid supplementation inhibited the expression of endoplasmic reticulum (ER) stress-specific proteins including GRP78, PERK, eIF2α, IRE1α, XBP1 and ATF6, subsequently decreasing the expression of proteins related to lipid synthesis including SREBP-1c, ACC1 and FAS. Conclusions: In conclusion, folic acid supplementation prior to and throughout pregnancy exerts preventive effects on high-fat diet-induced PPWR in rats, and the mechanism is associated with the inhibition of ER stress-mediated lipogenesis signaling pathways in the liver. Folic acid supplementation may serve as a potential strategy for preventing PPWR. In the future, the effectiveness of folic acid in PPWR prevention can be further verified by population studies.

## 1. Introduction

Postpartum weight retention (PPWR) refers to the disparity between body weight at a specified time post-delivery and body weight before pregnancy [1]. Although pre-pregnancy weight varies between women, nearly 50% of them retain a large proportion of pregnancy weight within 1 year postpartum [2]. It has been reported that as many as 20% of women retain more than 5 kg after 1 year postpartum [3]. A cohort study conducted in the US reported that almost 75% of women did not return to their pre-pregnancy weight 1 year postpartum, with 47.4% retaining over 4.5 kg and 24.2% retaining over 9.1 kg [4]. According to the Chinese National Nutrition and Health Surveillance, the prevalence of PPWR in China was 41.5%. The average weight retention at 3, 6 and 12 months after delivery was 5.3 kg, 4.5 kg and 3.1 kg, respectively [5]. Substantial PPWR has generally been considered to be retention of 5 kg or more after 1-year postpartum [6], which strongly predisposes women to a high risk for type 2 diabetes and long-term obesity [7,8]. Existing research has confirmed that factors associated with PPWR include pre-pregnancy body mass index, gestational weight gain (GWG), breastfeeding duration, dietary intake and physical activity [9,10,11]. Excessive GWG as the strongest predictor for PPWR is associated with higher energy intake during gestation [12]. A high-fat diet during gestation is a common occurrence and has been shown in population studies from multiple countries, including China, the United States and other western countries [13,14,15]. A high-fat diet during gestation reprograms maternal energy metabolism and increases GWG, leading to long-term postpartum obesity [16,17]. In recent years, limited studies have found that appropriate dietary interventions during gestation, such as vitamin D_3_, myo-inositol and fiber, could help to reduce excessive GWG and further improve PPWR [18,19,20].

Folic acid, one of the B complex vitamins, has received considerable attention owing to its role in the prevention of fetal neural tube defects [21]. Nowadays, accumulating studies have demonstrated some other positive effects of folic acid intake during gestation on the health of kids, such as reducing the risk of hepatic steatosis, congenital heart defects and autism spectrum disorders [22,23]. Furthermore, the role of folic acid supplementation prior to and throughout gestation in maternal health has been also well established [24,25]. For example, it was found that periconceptional folic acid supplementation was related to reduced risks of gestational diabetes mellitus and spontaneous preterm birth [24,25]. A recent study indicated that the intake of folic acid at a low dose (0 mg/kg) or a high dose (10 mg/kg or 20 mg/kg) during gestation resulted in delayed weight loss after giving birth [26]. However, it is unknown whether folic acid supplementation based on a tolerable intake level (5 mg/kg) can alleviate excessive GWG induced by a high-fat diet and thus improve PPWR, and its underlying molecular mechanism needs to be explored.

Endoplasmic reticulum (ER) is the primary site of lipid biosynthesis, where numerous enzymes and regulatory proteins of lipid metabolism exist [27]. A series of stress conditions such as hypoxia, glucose deprivation, inflammation and perturbation in Ca^2+^ homeostasis can increase the accumulation of unfolded/misfolded proteins in the ER, causing a stress situation termed ER stress [28,29]. A high-fat diet was found to alter ER calcium homeostasis and interfere with protein folding in the liver, resulting in ER stress [30,31]. ER stress could trigger the unfolded protein response (UPR), a signal transduction pathway that is mediated by three ER transmembrane sensor proteins including protein kinase RNA-like endoplasmic reticulum kinase (PERK), inositol-requiring enzyme 1α (IRE1α) and activating transcription factor 6 (ATF6). Under normal physiological conditions, a molecular chaperone in the ER lumen named glucose-regulated protein 78 (GRP78) binds to PERK, IRE1α and ATF6. In times of ER stress, GRP78 is preferentially recruited to unfolded/misfolded proteins and dissociates from the three ER stress sensors, contributing to activation of the UPR [32]. After dissociation from GRP78, PERK undergoes an autophosphorylation reaction and activates its downstream target protein eukaryotic initiation factor 2α (eIF2α) [33]. The activation of IRE1α under ER stress facilitates X box-binding protein-1 (XBP1) mRNA splicing, generating the spliced XBP1 (XBP1s) that enter the nucleus to initiate the transcription of genes associated with the UPR [29]. Upon dissociation of ATF6 from GRP78, it is transferred from the ER to the Golgi, where it is processed into its active form and translocated to the nucleus as a transcription factor [29]. An overactivated UPR under chronic ER stress conditions could initiate a series of metabolic disorders. Research has confirmed that ER stress promotes de novo lipogenesis by upregulating the expression of key lipogenesis markers through the UPR, which contributes to the progression of obesity [29].

Folic acid has been proven to regulate GRP78, which is involved in ER stress signaling, in both in vivo and in vitro studies [34,35,36]. However, whether folic acid supplementation can mitigate PPWR associated with excessive GWG by ameliorating ER stress remains elusive. In this study, rats were given 5 mg/kg folic acid to observe its preventive effect on PPWR induced by a high-fat diet and mainly to investigate the mechanism of its involvement in lipogenesis via ER stress.

## 2. Materials and Methods

### 2.1. Animal Experimental Design

Eight-week-old male and female *Sprague Dawley* rats weighing 250 ± 10 g were purchased from Beijing Vital River Laboratory Animal Technology Co., Ltd. (Beijing, China). The rats were reared in a specific-pathogen-free (SPF) environment maintained under a 12 h light-dark cycle at a temperature of 21–23 °C and a relative humidity of 40–60%. Following 1 week of acclimatization, female rats received random assignments to either a normal diet (2 mg folic acid/kg) or a folic acid-supplemented diet (5 mg folic acid/kg). All male rats were fed with a normal diet before mating. After 2 weeks, female rats were caged with male rats at a ratio of 3:1 for 12 h, and vaginal smears were taken from the female rats. The day when a large amount of sperm was observed under a microscope was recorded as gestational day (GD) 0.5. At GD0.5, the above two groups were then allocated to a normal diet or a high-fat diet. Thus, a total of 40 pregnant rats were assigned to four groups (*n* = 10 per group): the control group (CON), high-fat diet group (HF), control diet supplemented with folic acid group (FA) and high-fat diet supplemented with folic acid group (HF + FA). The compositions and energy contents of the experimental diets are summarized in Table S1. To reduce impact of differences in milk availability among the dams, pups were culled to six per litter within 24 h postpartum. After giving birth, all dams were given a normal diet. The body weight of each dam was monitored on a weekly basis. Food and water were abundantly accessible throughout the experiment. Water intake and food intake were measured daily throughout the experiment.

### 2.2. Sample Collection

Dams were sacrificed at 1 week PW, and blood samples were collected from the abdominal aorta, centrifuged at 3000 rpm for 10 min to obtain supernatant serum samples and ultimately preserved at −80 °C before analysis. The liver, perirenal fat, mesenteric fat and periovarian fat were rapidly removed from dams and weighed immediately. Total visceral fat represents the sum of perirenal, mesenteric and periovarian fat. The liver index and fat index were determined by calculating liver weights and fat weights as percentages of the final body weights of the dams. A portion of liver tissue was promptly preserved in a 10% formaldehyde solution for hepatic histological examination. The residual liver tissue and fat were frozen in liquid nitrogen and finally preserved at −80 °C for further research.

The animal experimental procedures were approved by the Animal Care and Use Committee of the Medical College of Qingdao University (approval No. QDU-AEC-2023371, approval date 12 June 2023) and were carried out in strict accordance with the institution’s guidelines.

### 2.3. Serum Biochemical Assays

The serum biochemical parameters of triglycerides (TGs) and total cholesterol (TC) were determined using an AU5400 automatic biochemical analyzer (Beckman, Los Angeles, CA, USA). Serum homocysteine (Hcy) was measured by high-performance liquid chromatography according to a previous method [37].

### 2.4. Liver Biochemical Assays

Liver tissue was homogenized and centrifuged at 3000 rpm and 4 °C for 10 min. The supernatant was obtained, and the concentrations of TGs, TC, malonaldehyde (MDA), superoxide dismutase (SOD), glutathione peroxidase (GSH-Px) and catalase (CAT) (Jiancheng Bioengineering Institute, Nanjing, China) were measured with corresponding ELISA kits.

### 2.5. Histopathological Analyses of Liver and Fat

The liver and periovarian fat from the same parts of the dams were fixed by immersion in 10% formaldehyde solution, embedded in paraffin and then sliced to 5 μm thickness serially utilizing a paraffin slicing machine. The sections were cleared in xylene, dehydrated in an ethanol gradient and subjected to hematoxylin and eosin (H&E) staining. Histopathological alterations in tissues were observed using a BX60 light microscope system (Olympus, Tokyo, Japan).

### 2.6. Western Blot Analysis

The hepatic protein expression levels of GRP78, PERK, phosphorylated PERK (*p*-PERK), eIF2α, phosphorylated eIF2α (*p*-eIF2α), IRE1α, phosphorylated IRE1α (*p*-IRE1α), XBP1, ATF6 (Santa Cruz Biotechnology, Santa Cruz, CA, USA), sterol regulatory element binding protein-1c (SREBP-1c), acetyl-CoA carboxylase-1 (ACC1) and fatty acid synthase (FAS) (Abcam, Cambridge, UK) were measured by Western blot analysis in accordance with our previous methods [38,39,40]. β-Actin (Abways, Shanghai, China) served as an internal reference protein. The corresponding secondary antibodies were obtained from ABclonal Biotechnology Co., Ltd. (Wuhan, China).

### 2.7. Statistical Analysis

Data are presented as the mean ± standard deviation (SD). Statistical analysis was conducted using SPSS 22.0 statistical software (SPSS, Chicago, IL, USA) and GraphPad Prism 8 (GraphPad Software, San Diego, CA, USA). Group comparisons were conducted using one-way analysis of variance (ANOVA), followed by Fisher’s LSD test to assess differences between two groups when the ANOVA provided a significant result. Results were deemed statistically significant at *p* < 0.05.

## 3. Results

### 3.1. Folic Acid Supplementation Altered the Body Weights of Dams and the Birth Weights of Pups

**Figure 1:**
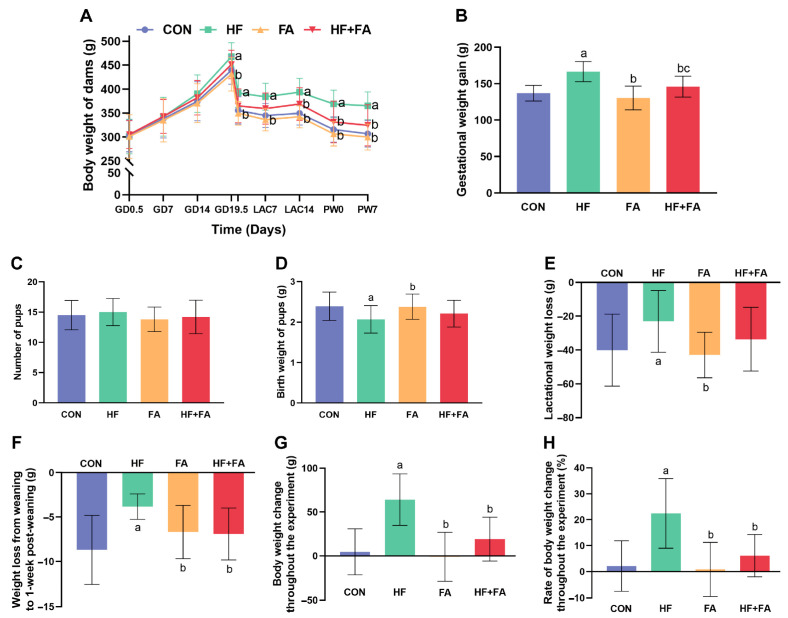
Folic acid supplementation altered the body weights of dams and the birth weights of pups. (**A**) Body weights of dams. (**B**) Gestational weight gain. (**C**) Numbers of pups. (**D**) Birth weights of pups. (**E**) Lactational weight loss. (**F**) Weight loss from weaning to 1 week post-weaning. (**G**) Body weight changes throughout the experiment. (**H**) Rates of body weight changes throughout the experiment. Values are presented as the mean ± SD (*n* = 10). ^a^ *p* < 0.05 indicates significant differences in comparison to the CON group. ^b^ *p* < 0.05 indicates significant differences in comparison to the HF group. ^c^ *p* < 0.05 indicates significant differences in comparison to the FA group.

The body weights of the dams exhibited no significant differences among the four groups at GD0.5 (*p* > 0.05) (Figure 1A). During gestation, the body weights of the dams in the four groups gradually increased. At GD19.5, the body weights of the dams in the HF group were significantly higher than those in the CON group (*p* < 0.05). GWG in the HF group was remarkedly higher than that in the CON group. In comparison to that in the HF group, supplementation with folic acid markedly lowered GWG induced by a high-fat diet (Figure 1B, *p* < 0.05). After giving birth, the body weights of the dams and pups were measured. The body weights of the dams in the HF group were still significantly higher than those in the CON group (Figure 1A, *p* < 0.05). Furthermore, no significant differences were found in number of pups among the four groups (Figure 1C, *p* > 0.05), while the birth weights of the pups in the HF group were significantly lower than those in the CON group (Figure 1D, *p* < 0.05). During lactation, body weights in all four groups decreased, but body weights in the HF group were always observably higher than those in the CON group (Figure 1A, *p* < 0.05). At LAC14 and LAC21 (PW0), the dams in the HF + FA group experienced significant weight loss in comparison to the dams in the HF group (Figure 1A, *p* < 0.05). Moreover, lactational weight loss in the HF group was noticeably lower than that in the CON group (Figure 1E, *p* < 0.05). After weaning, the body weights of the dams in each group further decreased. At PW7, the body weights of the dams in the HF group were remarkedly higher than those in the CON group. In comparison to that in the HF group, supplementation with folic acid significantly reduced the increase in body weight induced by a high-fat diet (Figure 1A, *p* < 0.05). From weaning to 1 week PW, weight loss in the HF group was significantly less than that in the CON group, and the HF + FA group had noticeably greater weight loss than the HF group (Figure 1F, *p* < 0.05). For the duration of the experiment, no significant differences in body weight changes were found between the CON group and the FA group. In comparison to those in the CON group, body weight changes and the rates of body weight changes among the dams revealed prominent increases in the HF group, while body weight changes and the rates of body weight changes among the dams in the HF + FA group were markedly decreased compared to those in the HF group (Figure 1G,H, *p* < 0.05).

### 3.2. Changes in Water Intake, Food Intake and Energy Intake

**Figure 2:**
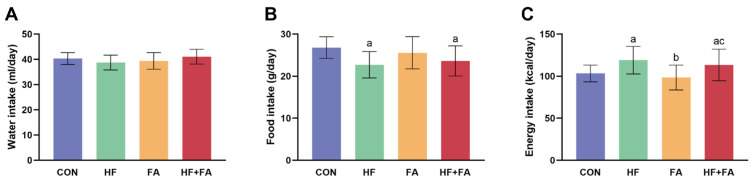
Changes in water intake, food intake and energy intake. (**A**) Water intake. (**B**) Food intake. (**C**) Energy intake. Values are presented as the mean ± SD (*n* = 10). ^a^ *p* < 0.05 indicates significant differences in comparison to the CON group. ^b^ *p* < 0.05 indicates significant differences in comparison to the HF group. ^c^ *p* < 0.05 indicates significant differences in comparison to the FA group.

There were no significant differences in water intake among the four groups (Figure 2A, *p* > 0.05). Food intake in the HF group and HF + FA group was significantly lower than that in the CON group (Figure 2B, *p* < 0.05). Conversely, the HF group and HF + FA group had significantly higher energy intake than the CON group (Figure 2C, *p* < 0.05). There were no significant differences in food intake and energy intake between the HF group and the HF + FA group.

### 3.3. Folic Acid Supplementation Ameliorated Serum Biochemical Profiles in Dams

**Figure 3:**
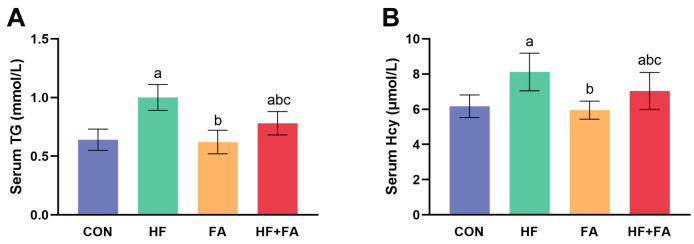
Folic acid supplementation ameliorated serum biochemical profiles in dams. (**A**) Serum TGs. (**B**) Serum Hcy. Values are presented as the mean ± SD (*n* = 10). ^a^ *p* < 0.05 indicates significant differences in comparison to the CON group. ^b^ *p* < 0.05 indicates significant differences in comparison to the HF group. ^c^ *p* < 0.05 indicates significant differences in comparison to the FA group.

The dams in the HF group showed significantly elevated serum TG levels compared with those in the CON group. Serum TG levels in the HF + FA group were observably decreased in comparison to those in the HF group (Figure 3A, *p* < 0.05). Compared with that in the CON group, serum TC in the HF group was significantly increased (Figure S1, *p* < 0.05). Serum Hcy in the HF group was significantly higher than that in the CON group, and serum Hcy in the HF + FA group was significantly lower than that in the HF group (Figure 3B, *p* < 0.05).

### 3.4. Folic Acid Supplementation Reduced Fat Weight and the Fat Index in Dams

**Figure 4:**
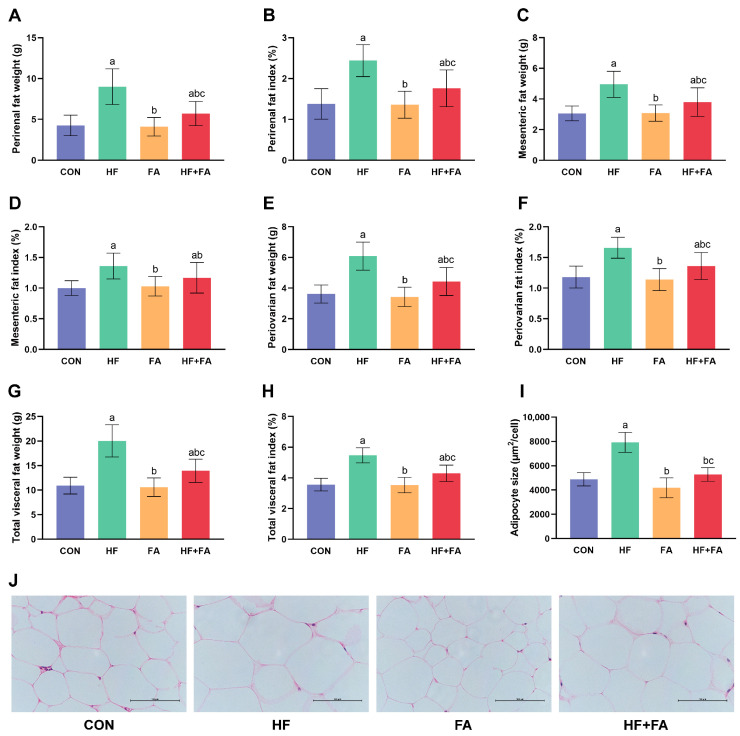
Folic acid supplementation reduced fat weight and the fat index in dams. (**A**) Perirenal fat weight. (**B**) Perirenal fat index. (**C**) Mesenteric fat weight. (**D**) Mesenteric fat index. (**E**) Periovarian fat weight. (**F**) Periovarian fat index. (**G**) Total visceral fat weight. (**H**) Total visceral fat index. (**I**) Adipocyte size. (**J**) Pathological changes in periovarian fat. H&E staining of perirenal fat was photographed at 400× magnification. Scale bar = 100 μm. Adipocyte size was measured by using ImageJ software (version 1.8.0). Values are presented as the mean ± SD (*n* = 10). ^a^ *p* < 0.05 indicates significant differences in comparison to the CON group. ^b^ *p* < 0.05 indicates significant differences in comparison to the HF group. ^c^ *p* < 0.05 indicates significant differences in comparison to the FA group.

The weights and indices of perirenal, mesenteric, periovarian and total visceral fat in the HF group were markedly higher than those in the CON group (Figure 4A–H, *p* < 0.05). Supplementation with folic acid significantly attenuated the increases in these parameters induced by a high-fat diet (Figure 4A–H, *p* < 0.05). Histopathological examination of periovarian fat revealed that the sizes of adipocytes in the HF group were significantly larger than those in the CON group. The numbers of cells within identical microscopic fields in the HF group were clearly reduced in comparison to those in the CON group. The HF + FA group showed a smaller adipocyte size and an elevated cell number within identical microscopic fields compared with the HF group (Figure 4I,J, *p* < 0.05).

### 3.5. Folic Acid Supplementation Reduced Liver Weight and the Liver Index and Ameliorated Hepatic Lipids Profiles in Dams

**Figure 5:**
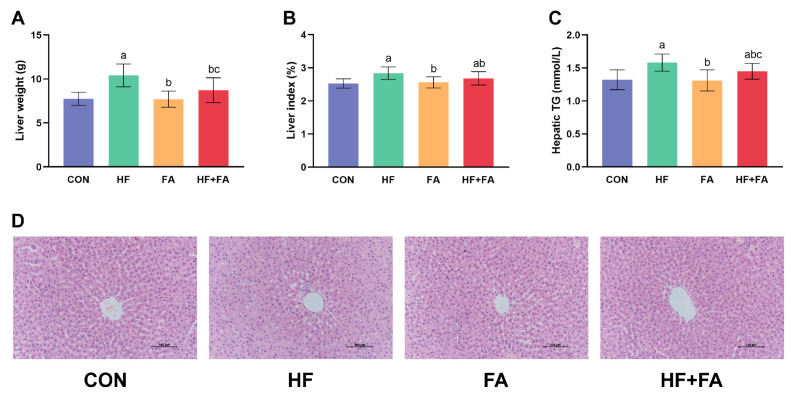
Folic acid supplementation reduced liver weight and the liver index and ameliorated hepatic lipids profiles in dams. (**A**) Liver weight. (**B**) Liver index. (**C**) Hepatic TGs. (**D**) Pathological changes in the liver. H&E staining of the liver tissue was photographed at 200 × magnification. Scale bar = 100 μm. Values are presented as the mean ± SD (*n* = 10). ^a^ *p* < 0.05 indicates significant differences in comparison to the CON group. ^b^ *p* < 0.05 indicates significant differences in comparison to the HF group. ^c^ *p* < 0.05 indicates significant differences in comparison to the FA group.

Liver weights and liver indices in the HF group were remarkedly higher than those in the CON group, while folic acid supplementation could obviously diminish the increases in liver weights and liver indices induced by a high-fat diet (Figure 5A,B, *p* < 0.05). Meanwhile, higher levels of TGs in the liver were prominent in the HF group compared to the CON group. Supplementation with folic acid observably reduced the elevated hepatic TG levels induced by a high-fat diet (Figure 5C, *p* < 0.05). Compared with those in the CON group, hepatic TC levels were significantly increased in the HF group. Hepatic TC levels were significantly lower in the HF + FA group than in the HF group (Figure S1, *p* < 0.05). H&E staining of the liver was performed to visualize histopathological differences among the four groups. Liver sections in the CON group and FA group showed a clear and complete lobular structure, with hepatic cords around the central vein arranged radially. The hepatic cords in the HF group were disordered, with notable lipid vacuoles and slight infiltration of inflammatory cells surrounding the central vein. Compared with observations in the HF group, the number of lipid vacuoles significantly decreased, and no apparent inflammatory cell infiltration was observed in the HF + FA group (Figure 5D).

### 3.6. Folic Acid Supplementation Alleviated Hepatic Oxidative Stress in Dams

**Figure 6:**
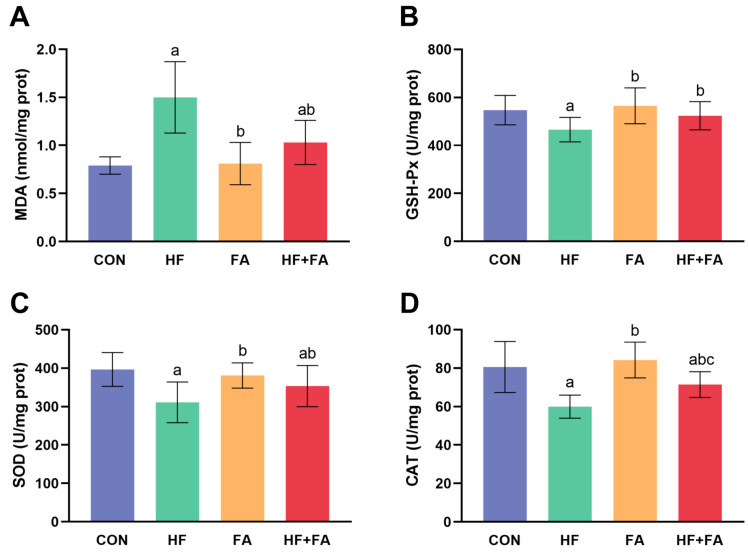
Folic acid supplementation alleviated hepatic oxidative stress in dams. (**A**) Hepatic MDA. (**B**) Hepatic GSH-Px. (**C**) Hepatic SOD. (**D**) Hepatic CAT. Values are presented as the mean ± SD (*n* = 10). ^a^ *p* < 0.05 indicates significant differences in comparison to the CON group. ^b^ *p* < 0.05 indicates significant differences in comparison to the HF group. ^c^ *p* < 0.05 indicates significant differences in comparison to the FA group.

The levels of MDA, GSH-Px, SOD and CAT exhibited no significant differences between the CON group and the FA group (*p* > 0.05) (Figure 6). In comparison to those in the CON group, hepatic MDA levels in the HF group were observably increased, while hepatic GSH-Px, SOD and CAT levels were significantly decreased (*p* < 0.05). In comparison to those in the HF group, hepatic MDA levels in the HF + FA group were significantly decreased, while hepatic GSH-Px, SOD and CAT levels were noticeably increased (*p* < 0.05).

### 3.7. Folic Acid Supplementation Alleviated Hepatic ER Stress and Reduced Lipid Synthesis in Dams

**Figure 7:**
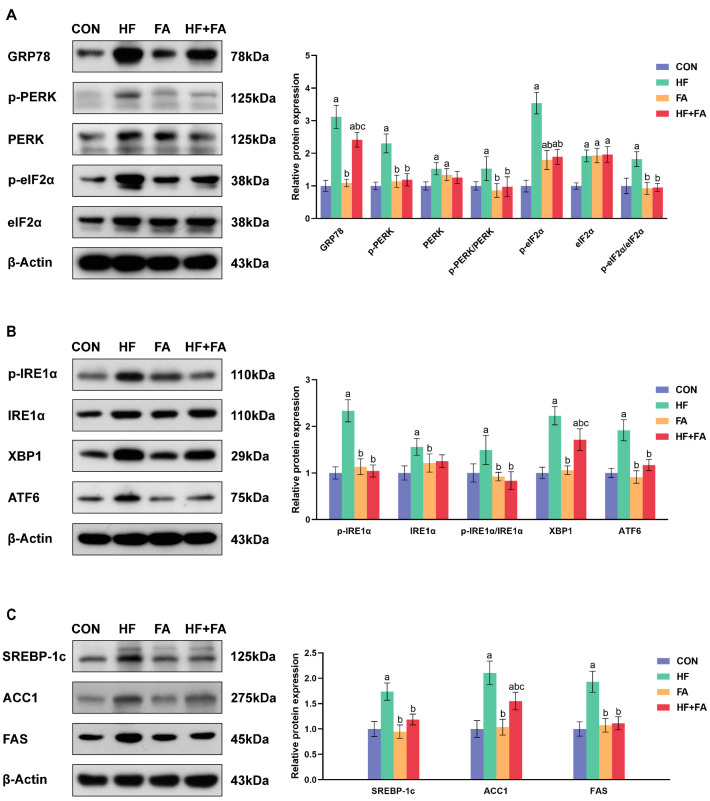
Folic acid supplementation alleviated hepatic ER stress and reduced lipid synthesis in dams. (**A**,**B**) Protein expression levels related to ER stress. (**C**) Protein expression levels related to lipid synthesis. Values are presented as the mean ± SD (*n* = 3). ^a^ *p* < 0.05 indicates significant differences in comparison to the CON group. ^b^ *p* < 0.05 indicates significant differences in comparison to the HF group. ^c^ *p* < 0.05 indicates significant differences in comparison to the FA group.

In comparison to those in the CON group, the expression levels of GRP78, *p*-PERK/PERK and *p*-eIF2α/eIF2α showed significant increases in the HF group (*p* < 0.05). Supplementation with folic acid prominently lowered the elevated expression levels of these indices induced by a high-fat diet (*p* < 0.05) (Figure 7A). Moreover, the expression levels of *p*-IRE1α/IRE1α, XBP1 and ATF6 in the HF group were significantly higher than those in the CON group (*p* < 0.05). Supplementation with folic acid significantly reduced the elevated expression levels of *p*-IRE1α/IRE1α, XBP1 and ATF6 induced by a high-fat diet (*p* < 0.05) (Figure 7B). The expression levels of lipid synthesis-related proteins including SREBP-1c, ACC1 and FAS in the HF group were remarkably higher than those in the CON group (*p* < 0.05). Supplementation with folic acid significantly decreased the elevated expression levels of these proteins induced by a high-fat diet (*p* < 0.05) (Figure 7C).

## 4. Discussion

**Figure 8:**
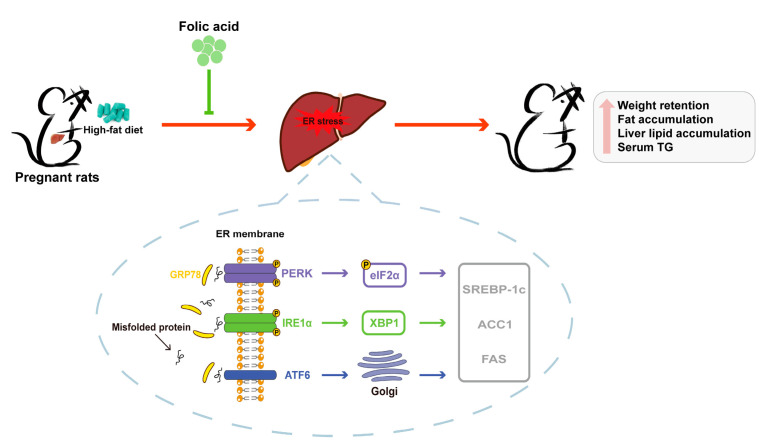
Potential mechanism underlying the preventative effect of folic acid on PPWR.

In this study, we elaborated the preventive effect of folic acid against PPWR and explored its potential mechanism. Specifically, folic acid supplementation may attenuate high-fat diet-induced hepatic ER stress by suppressing GRP78-mediated PERK-eIF2α, IRE1α-XBP1 and ATF6 signaling pathways, thereby downregulating the expression of downstream lipogenic genes and ultimately alleviating PPWR in rats (Figure 8).

Pregnancy, as an occurrence of biology, can trigger physiological alterations in body composition. As pregnancy progresses, the weights of products of conception (the placenta, fetus and amniotic fluid) increase, along with changes in maternal physiology, including the deposition of protein, fat and water in the uterus, mammary glands and blood, which thus become components of GWG [41]. To ensure adequate energy stores for maternal consumption and fetal growth, women may increase their food intake during gestation. However, blindly increasing food intake can lead to an unreasonable dietary structure and excess energy intake. Intake of a high-fat diet during gestation is common and has been found in many population studies [13,14,15]. High-fat diet intake during gestation induces increased body fat and excessive GWG, which is one of the main causes of PPWR [9,16]. After parturition, maternal body weight gradually declines, and maternal metabolism starts to return to the pre-pregnancy setpoint. Lactation is regarded as a critical process that accelerates the reset of maternal metabolism to homeostasis [42]. As previously reported, the 1-week PW time point was selected to observe the reset of maternal metabolism and increases in body weight [26]. Therefore, we dynamically monitored body weight changes in dams from GD 0.5 to 1 week PW to evaluate PPWR. We found that the HF group had greater GWG, which corresponds to findings in previous studies [17,43]. Given that the numbers and weights of pups were notable factors affecting the weights of pregnant rats, we measured the numbers and birth weights of pups. No significant differences were observed in the number of pups according to our findings. Nevertheless, the birth weights of the pups in the HF group were prominently lower than those in the CON group, which may be attributed to high-fat diet-induced fetal intrauterine growth restriction [38]. The aforementioned results suggest that the increased body weights of the pregnant rats in the HF group may be mainly caused by excessive accumulation of maternal fat. During lactation, body weights in the HF group decreased but remained markedly elevated compared with those in the CON group at each time point. Weight loss in the HF group during lactation was notably less than that in the CON group. After weaning, body weights in the HF group continued to decrease, but the loss was still significantly less than that in the CON group. To exclude the impact of a dam’s own body weight on the results, we calculated the rate of body weight changes during the experimental period to evaluate PPWR and found that the rate of body weight changes in the HF group was obviously higher than that in the CON group. Visceral fat accumulation is another important indicator of PPWR because adipose tissue preferentially deposits in the visceral compartment as pregnancy progresses [44]. In this study, visceral fat weights in the HF + FA group were significantly lower than those in the HF group. The sizes of adipocytes in the HF + FA group were significantly smaller than those in the HF group. The above results indicated that high-fat diet intake during gestation resulted in PPWR in dams.

Population studies have shown that the serum folate level is closely related to body weight [45,46]. Overweight or obese women usually have lower serum folate levels than women of normal weights [45]. Studies have revealed that folic acid is involved in the process of lipid metabolism [47,48,49]. Chronic folic acid deficiency may induce hepatic steatosis by disrupting choline metabolism in mice [47]. Dietary folic acid supplementation could inhibit high-fat diet-induced obesity and improve lipid metabolism in mice [48,49]. Recent research indicated that folic acid intake below requirements during gestation delayed the reset of maternal metabolism and resulted in PPWR in dams [26]. However, whether folic acid supplementation prevents PPWR induced by a high-fat diet during gestation has not been documented. The content of folic acid in normal feed is 2 mg/kg, which is commonly used to reflect the recommended intake level in human diets [50,51]. A folic acid supplement dose of 5 mg/kg has been widely used in animal studies to show its biological functions. Research has demonstrated that 5 mg/kg folic acid supplementation had an inhibitory role in body weight gain, affecting the expression of genes associated with fat and energy metabolism in high-fat diet-fed mice [48,49,52]. Thus, dams fed a high-fat diet were given 2 mg/kg or 5 mg/kg folic acid in this study, and variations in the body weights of dams were observed from GD 0.5 to 1 week PW. In this study, no significant difference was identified in body weight between the FA group and the CON group throughout the experimental period, suggesting that the dose of 5 mg/kg folic acid supplementation had no adverse effect on the body weights of dams. Our results revealed that weight gain during gestation was remarkedly reduced in the HF + FA group compared with the HF group. After childbirth, the body weights of the dams in the HF group and HF + FA group started to decrease. From two weeks postpartum, maternal body weights in the HF + FA group were significantly lower than those in the HF group. Weight loss between weaning and 1 week PW was considerably greater in the HF + FA group than in the HF group. In comparison to the HF group, the HF + FA group had a lower rate of body weight changes throughout the experiment. Visceral fat weights in the HF + FA group were significantly lower than those in the HF group. The above results confirmed the preventive effect of folic acid supplementation pre-pregnancy and during pregnancy on PPWR induced by a high-fat diet in dams. Given that dietary intake is a notable factor affecting body weight, we analyzed the levels of water intake, food intake and energy intake among the groups. We found that water intake was similar in the four groups. There was no statistically significant difference in food intake or energy intake between the HF group and the HF + FA group, suggesting that the preventive effect of 5 mg/kg folic acid supplementation on PPWR is not achieved by affecting appetite and energy intake.

Previous animal studies found that the mechanism by which folic acid supplementation diminishes elevated body weight induced by a high-fat diet may be related to alterations in mitochondrial biogenesis-associated genes in adipose tissue and gut microbiota-associated branched-chain amino acids [48,49]. A high-fat diet can cause ER stress, which has been found in numerous in vivo studies [53,54]. Through in vitro experiments, high-fat intake has been proven to alter ER calcium homeostasis and disturb ER proteostasis in the liver [30,31]. When unfolded or misfolded proteins accumulate in the ER, ER stress is triggered [31]. ER stress induced by a high-fat diet can lead to increased hepatic lipogenesis, resulting in increased serum TG levels and increased visceral adipose tissue, ultimately increasing body weight gain [53,55,56]. It has not been investigated whether folic acid supplementation can alleviate hepatic ER stress caused by a high-fat diet and thus have a preventive effect on PPWR. As an integral ER stress sensor, GRP78 binds to PERK, IRE1α and ATF6 under physiological conditions. Under high-fat diet-induced stress, GRP78 dissociates from the three ER stress sensors and activates corresponding downstream substrates, inducing ER stress [32,57]. The regulation of folic acid on GRP78 has been well demonstrated in in vitro studies [35,36]. For example, a folic acid intervention inhibited the upregulation of GRP78 and attenuated Hcy-induced ER stress in MC3T3-E1 cells [35]. Additionally, folic acid treatment increased the expression of GRP78 and reversed the decline in the ER stress response in a doxorubicin-induced senescence model of H9C2 cardiomyocytes [36]. Therefore, it is reasonable to speculate that folic acid can alleviate ER stress by regulating GRP78, thereby improving lipid synthesis. In this study, we measured the expression levels of proteins associated with hepatic ER stress and found that the expression levels of GRP78 and its downstream PERK, eIF2α, IRE1α, XBP1 and ATF6 were downregulated in the HF + FA group compared with the HF group. The results suggested that folic acid supplementation can inhibit ER stress induced by a high-fat diet.

Hepatic lipogenesis is one of the critical processes of lipid metabolism and is closely related to the occurrence of obesity [58]. ER stress affects lipid homeostasis by regulating the expression of a series of transcription factors. SREBP-1c is a key transcription factor that regulates hepatic de novo lipogenesis [59], which regulates the expression of hepatic lipogenic genes such as ACC1 and FAS [60]. ACC1 and FAS act as two step-limiting enzymes for de novo lipogenesis. ACC1 facilitates the conversion of acetyl-CoA into malonyl-CoA. Malonyl-CoA is subsequently transformed into palmitic acid by FAS. Finally, palmitic acid undergoes elongation and desaturation reactions to generate monounsaturated fatty acids, which are the main fatty acid components of TGs [61,62]. Accumulating evidence implies that activation of SREBP-1c and subsequent lipogenesis are secondary to ER stress. Specifically, ER stress could increase the expression of the mature form of SREBP-1c in the nucleus by inducing cleavage of its precursor form. The PERK-eIF2α signaling pathway enhances the expression of SREBP-1c, ACC1 and FAS, thereby aggravating lipid deposition in calf hepatocytes [63]. Knockdown of the XBP1 gene by small interfering RNA prevents activation of the SREBP-1c promoter in hepatoma cells, indicating that SREBP-1c is involved in the process of the IRE1α-XBP1 pathway promoting hepatic lipogenesis [64]. Knockdown of ATF6α can decrease the mRNA expression of SREBP-1c and inhibit the differentiation of an adipogenic cell line into mature adipocytes, suggesting the role of ATF6 in the lipogenic process [65]. Therefore, proteins involved in lipid synthesis regulated by ER stress were detected in the liver in this study, including SREBP-1c, ACC1 and FAS. We discovered that with the improvement in ER stress caused by folic acid supplementation, the expression levels of SREBP-1c, ACC1, and FAS were downregulated in the liver. The results indicated that folic acid supplementation can reduce de novo lipogenesis by ameliorating hepatic ER stress, thereby preventing the occurrence of PPWR.

A high-fat diet may increase the level of Hcy in the circulation by downregulating the expression of cystathionine-β-synthase and cystathionine-γ-lyase in the liver [66]. Elevated circulating Hcy can regulate the expression of genes involved in hepatic lipogenesis in hepatocytes and induce lipid accumulation via activation of transcription factors [67,68]. Eliminating Hcy can inhibit the activity of activator protein 1 and its induction of FAS gene transcription while inhibiting lipid synthesis in hepatocytes [69]. Dietary folic acid can be metabolized to 5-methyltetrahydrofolate in vivo. Furthermore, 5-methyltetrahydrofolate is used as a methyl donor, with vitamin B_12_ as an essential cofactor, facilitating the conversion of Hcy to methionine [70]. Combined folic acid and vitamin B_12_ supplementation can significantly reduce Hcy levels [71,72]. Even with folic acid supplementation alone, its effect on reducing Hcy levels has been well validated in numerous human and animal experiments [73,74]. Hence, serum Hcy levels in the rats in each group were measured in this study. We found that serum Hcy was higher in the HF group than in the CON group, and serum Hcy was lower in the HF + FA group than in the HF group. The mechanism of the preventive effect of folic acid supplementation on high-fat diet-induced PPWR in this study may be also related to its regulation of Hcy. In addition, folic acid supplementation could increase *n*-3 polyunsaturated fatty acids levels in plasma [75], and the elevation of *n*-3 polyunsaturated fatty acids could improve disorder of hepatic lipid metabolism by inhibiting hepatic ceramide synthesis [76]. Therefore, the mechanism by which folic acid supplementation prevents high-fat diet-induced PPWR cannot be ruled out to be mediated by affecting other nutrients levels (such as *n*-3 polyunsaturated fatty acids). This study is the first to explore the potential mechanism of folic acid in preventing PPWR induced by a high-fat diet. In addition to ER stress-mediated hepatic lipid synthesis, more potential mechanisms need to be uncovered in future studies.

Some limitations of this study should be noted. Firstly, the results of this study are based on animal models, and extrapolation of the research findings is limited due to species differences between rats and humans. In the future, the effectiveness of folic acid in PPWR prevention can be further verified by population studies. Secondly, the dietary intervention used in this study is one of the common methods of folic acid supplementation in animal studies due to its low irritation, but this method could not guarantee that the rats in each group consumed the same amount of folic acid. During the dietary intervention period, we provided sufficient food to the rats to avoid restricted folic acid intake due to food shortages.

## 5. Conclusions

The effects of folic acid supplementation on maternal health during pregnancy and offspring health after birth have been extensively studied. However, limited research has been conducted on the effects of folic acid supplementation pre-pregnancy and during pregnancy on maternal postpartum health, especially in terms of body weight. This study offers the first evidence that folic acid supplementation has preventive effects against high-fat diet-induced PPWR in rats, and its mechanism was associated with a reduction in ER stress-mediated hepatic lipogenesis. This study provides a potential strategy for the prevention of PPWR.

## Data Availability

The data presented in this study are available on request from the corresponding author. The data are not publicly available due to laboratory policies and confidentiality agreements.

## Supplementary Materials

The following supporting information can be downloaded at: https://www.mdpi.com/article/10.3390/nu16244377/s1, Table S1: Compositions and energy contents of the experimental diets. Figure S1: Effects of folic acid supplementation on serum TC and hepatic TC in dams.

## Abbreviations

ACC1	acetyl-CoA carboxylase-1
ATF6	activating transcription factor 6
CAT	catalase
eIF2α	eukaryotic initiation factor 2α
ER	endoplasmic reticulum
FAS	fatty acid synthase
GRP78	glucose-regulated protein 78
GSH-Px	glutathione peroxidase
GWG	gestational weight gain
Hcy	homocysteine
H&E	hematoxylin and eosin
IRE1α	inositol-requiring enzyme 1α
MDA	malonaldehyde
PERK	protein kinase RNA-like endoplasmic reticulum kinase
PPWR	postpartum weight retention
PW	post-weaning
*p*-eIF2α	phosphorylated eukaryotic initiation factor 2α
*p*-IRE1α	phosphorylated inositol-requiring enzyme 1α
*p*-PERK	phosphorylated protein kinase RNA-like endoplasmic reticulum kinase
SOD	superoxide dismutase
SREBP-1c	sterol regulatory element binding protein-1c
TC	total cholesterol
TG	triglyceride
UPR	unfolded protein response
XBP1	X box-binding protein-1
