# Supplementary material for: Folic Acid Prevents High-Fat Diet-Induced Postpartum Weight Retention in Rats, Which Is Associated with a Reduction in Endoplasmic Reticulum Stress-Mediated Hepatic Lipogenesis
Source: Nutrients. 2024 Dec 19;16(24):4377. doi: 10.3390/nu16244377 (PMC11676124; doi:10.3390/nu16244377)
Supplement: Supplementary file 1 [file nutrients-16-04377-s001.zip › Supplementary Materials/Figure S1.pdf]

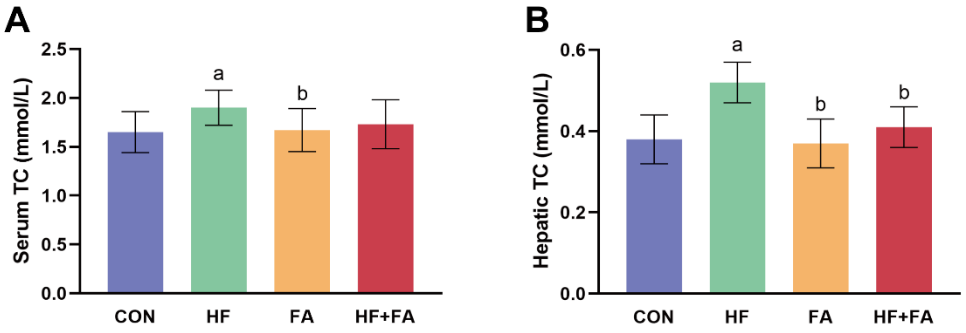

Figure S1. Folic acid supplementation ameliorates serum and hepatic TC in dams. (A) Serum TC. (B) Hepatic TC. [a] $p < 0.05$ represents significant differences in comparison to CON group. [b] $p < 0.05$ represents significant differences in comparison to HF group.
