# Supplementary material for: Folic Acid Prevents High-Fat Diet-Induced Postpartum Weight Retention in Rats, Which Is Associated with a Reduction in Endoplasmic Reticulum Stress-Mediated Hepatic Lipogenesis
Source: Nutrients. 2024 Dec 19;16(24):4377. doi: 10.3390/nu16244377 (PMC11676124; doi:10.3390/nu16244377)
Supplement: Supplementary file 1 [file nutrients-16-04377-s001.zip › Supplementary Materials/Table S1.pdf]

**Table S1.** Composition and energy of the experimental diets.

| Diet component | CON | HF | FA | HF+FA |
|---|---|---|---|---|
| **Ingredients** | | | | |
| Casein (g/1055g) | 200 | 200 | 200 | 200 |
| Corn starch (g/1055g) | 506.2 | - | 506.2 | - |
| Maltodextrin (g/1055g) | 125 | 125 | 125 | 125 |
| Sucrose (g/1055g) | 68.8 | 68.8 | 68.8 | 68.8 |
| Cellulose (g/1055g) | 50 | 50 | 50 | 50 |
| Lard (g/1055g) | 20 | 245 | 20 | 245 |
| Soybean oil (g/1055g) | 25 | 25 | 25 | 25 |
| Cystine (g/1055g) | 3 | 3 | 3 | 3 |
| Mineral mix (g/1055g) | 10 | 10 | 10 | 10 |
| Vitamin Mix Without Folic Acid (g/1055g) | 10 | 10 | 10 | 10 |
| Choline (g/1055g) | 2 | 2 | 2 | 2 |
| Limestone ore powder (g/1055g) | 5.5 | 5.5 | 5.5 | 5.5 |
| Calcium hydrophosphate (g/1055g) | 13 | 13 | 13 | 13 |
| Potassium citrate monohydrate (g/1055g) | 16.5 | 16.5 | 16.5 | 16.5 |
| Folic acid (mg/1055g) | 2.11 | 1.55 | 5.275 | 3.87 |

| Energy source | | | | |
|---|---|---|---|---|
| Energy from fat (%) | 10 | 60 | 10 | 60 |
| Energy from carbohydrate (%) | 70 | 20 | 70 | 20 |
| Energy from protein (%) | 20 | 20 | 20 | 20 |
| Energy (kcal/kg) | 3850 | 5243 | 3850 | 5243 |
